# YAP1 is an independent prognostic marker in pancreatic cancer and associated with extracellular matrix remodeling

**DOI:** 10.1186/s12967-020-02254-7

**Published:** 2020-02-13

**Authors:** Qimin Zhou, Monika Bauden, Roland Andersson, Dingyuan Hu, György Marko-Varga, Jianfeng Xu, Agata Sasor, Hua Dai, Krzysztof Pawłowski, Katarzyna Said Hilmersson, Xi Chen, Daniel Ansari

**Affiliations:** 1 The Eye Hospital, School of Ophthalmology and Optometry, Wenzhou Medical University, Wenzhou, Zhejiang China; 2 Department of Surgery, Clinical Sciences Lund, Lund University and Skåne University Hospital, 221 85 Lund, Sweden; 3 Clinical Protein Science and Imaging, Biomedical Centre, Department of Biomedical Engineering, Lund University, Lund, Sweden; 4 Department of Surgery, The Second Affiliated Hospital and Yuying Children’s Hospital of Wenzhou Medical University, Wenzhou, Zhejiang China; 5 Department of Pathology, Skåne University Hospital, Lund, Sweden; 6 Department of Pathology, The First Affiliated Hospital of Nanchang University, Nanchang, China; 7 Department of Experimental Design and Bioinformatics, Warsaw University of Life Sciences, Warsaw, Poland; 8 Department of Translational Medicine, Lund University, Malmö, Sweden

**Keywords:** Pancreatic cancer, YAP1, Transcriptomics, Proteomics, Prognosis, Extracellular matrix remodeling, Cancer

## Abstract

**Background:**

Pancreatic cancer is a major cause of cancer-related mortality. The identification of effective biomarkers is essential in order to improve management of the disease. Yes-associated protein 1 (YAP1) is a downstream effector of the Hippo pathway, a signal transduction system implicated in tissue repair and regeneration, as well as tumorigenesis. Here we evaluate the biomarker potential of YAP1 in pancreatic cancer tissue.

**Methods:**

YAP1 was selected as a possible biomarker for pancreatic cancer from global protein sequencing of fresh frozen pancreatic cancer tissue samples and normal pancreas controls. The prognostic utility of YAP1 was evaluated using mRNA expression data from 176 pancreatic cancer patients in The Cancer Genome Atlas (TCGA), as well as protein expression data from immunohistochemistry analysis of a local tissue microarray (TMA) cohort comprising 140 pancreatic cancer patients. Ingenuity Pathway Analysis was applied to outline the interaction network for YAP1 in connection to the pancreatic tumor microenvironment. The expression of YAP1 target gene products was evaluated after treatment of the pancreatic cancer cell line Panc-1 with three substances interrupting YAP–TEAD interaction, including Super-TDU, Verteporfin and CA3.

**Results:**

Mass spectrometry based proteomics showed that YAP1 is the top upregulated protein in pancreatic cancer tissue when compared to normal controls (log2 fold change 6.4; p = 5E−06). Prognostic analysis of YAP1 demonstrated a significant correlation between mRNA expression level data and reduced overall survival (p = 0.001). In addition, TMA and immunohistochemistry analysis suggested that YAP1 protein expression is an independent predictor of poor overall survival [hazard ratio (HR) 1.870, 95% confidence interval (CI) 1.224–2.855, p = 0.004], as well as reduced disease-free survival (HR 1.950, 95% CI 1.299–2.927, p = 0.001). Bioinformatic analyses coupled with in vitro assays indicated that YAP1 is involved in the transcriptional control of target genes, associated with extracellular matrix remodeling, which could be modified by selected substances disrupting the YAP1-TEAD interaction.

**Conclusions:**

Our findings indicate that YAP1 is an important prognostic biomarker for pancreatic cancer and may play a regulatory role in the remodeling of the extracellular matrix.

## Background

Pancreatic cancer is one of the most aggressive malignancies with a dismal 5-year survival rate of 9% [1]. It has surpassed breast cancer to become the third leading cause of cancer-related death and is estimated to rise to the second leading cause by 2030 [2]. Multiple factors, such as late diagnosis and resistance to conventional therapies, contribute to the overall poor prognosis.

The ability to identify subgroups of patients that may benefit from specific clinical management is considered central to modern precision oncology. For that purpose, large-scale genomic studies have been performed to determine molecular subtypes of pancreatic cancer requiring individualized treatments [3–6]. Such studies have massively increased our understanding of pancreatic cancer at the molecular level.

Proteomics is a valuable complement to genetic studies. Mass spectrometry (MS)-based proteomics profiling of patient-derived samples has been suggested as an effective approach for the discovery of biomarkers and detection of suitable therapeutic targets in many cancers [7–10].

Yes-associated protein 1 (YAP1) is a downstream effector of the Hippo signaling pathway, which is involved in tissue repair and regeneration, as well as tumorigenesis. Activation of the Hippo pathway leads to inactivation of YAP1 by cytoplasmic retention or proteolytic degradation [11, 12]. YAP1 in its active form, on the other hand, functions as a transcriptional co-activator predominantly mediated by an interaction with TEAD transcription factors [13]. Active YAP1 is also recognized as a potent oncogene closely linked to the progression of several cancer types [14, 15]. However, the role of the YAP1-TEAD interaction in regulating the expression of target genes in pancreatic cancer has not been completely explored.

In a previous study [10], we identified YAP1 as a differentially expressed protein between pancreatic cancer and normal controls using MS-based proteomics profiling. In the present study, we investigate the prognostic utility and the biological significance of YAP1 in pancreatic cancer using large and clinically well-annotated cohorts, complemented by bioinformatics and in vitro experimental analyses.

## Materials and methods

### Patient samples

For the MS-based proteomics, fresh frozen pancreatic cancer tissues (n = 10) were collected from patients with pancreatic ductal adenocarcinoma undergoing pancreaticoduodenectomy between July 2013 and April 2015 at the Department of Surgery, Skåne University Hospital, Lund, Sweden. Written informed consent was obtained from the patients included in the study. Age and gender-matched, fresh frozen, normal pancreatic biopsies (n = 10) were assessed from organ donors and obtained from the national consortium Excellence of Diabetes Research in Sweden and Lund University Diabetes center (LUDC).

The immunohistochemical (IHC) target verification was performed using tissue microarrays (TMA) from archival formalin-fixed paraffin-embedded (FFPE) resection specimens from 140 patients with pancreatic ductal adenocarcinoma who underwent curative intent pancreatic surgery from 1995 to 2017 at Skåne University Hospital, Lund and Malmö, Sweden.

All samples were histopathologically verified and selected by a specialized surgical pathologist prior to analysis. Ethical permission for the study was granted by the Ethical Committee at Lund University (Ref 2010/684, 2012/661, 2015/266, 2017/320). The REMARK guidelines were followed where applicable [16].

### MS-based proteomics

Sample processing and LC–MS/MS analysis were performed as reported previously [10]. Briefly, proteins extracted from fresh frozen pancreas specimens were reduced, alkylated and digested into peptides using Lys-C and trypsin. The peptides were analyzed using a high-performance liquid chromatography system, EASY-nLC 1000 connected to Q Exactive quadrupole-Orbitrap mass spectrometer equipped with a nanospray ion source (Thermo-Fisher Scientific, Bremen, Germany). To identify the detected proteins, the acquired MS/MS data were managed using Proteome Discoverer software, version 1.4 (Thermo Fisher).

### mRNA expression data

Publicly available transcriptomics data were retrieved from 176 pancreatic cancer patients from The Cancer Genome Atlas (TCGA) [17–19]. RNA-seq data were analyzed as the number of Fragments Per Kilobase of exon per Million reads (FPKM).

### Tissue microarray

The TMA was constructed from FFPE pancreatic tumors by a trained biomedical technician using an automated tissue array device (Minicore^®^ 3, Alphelys, Plaisir, France). A set of 4 cores with a diameter of 2 mm were extracted from each specimen and fixed into a new paraffin block. The completed blocks were then sectioned into 3 µm thick sections and mounted on glass slides.

### Immunohistochemistry

IHC analysis was performed as described previously [20]. Briefly, deparaffinization, rehydration and antigen-retrieval were performed using the automated PT Link system (Dako, Agilent Technologies, Glostrup, Denmark). TMA-slides were then incubated with monoclonal rabbit anti-human primary antibody against YAP1 (dilution 1:200; Cell Signaling) followed by biotinylated goat anti-rabbit secondary antibody (dilution 1:200; Vector Laboratories, Burlingame, CA). Avidin–biotin–peroxidase complex (Vectastain Elite ABC-HRP Kit, Vector Laboratories, Burlingame, CA) was used for signal amplification. The color was developed using chromogen diaminobenzidine (DAB) (Vector Laboratories). The nuclei were colored with hematoxylin. The immunostaining was evaluated by three independent pathologists, blinded to clinical information. H-score was applied as a semiquantitative approach [21, 22]. The intensity of YAP1 staining was scored as [0] (negative), [1+] (weak), [2+] (moderate), or [3+] (strong) and the percentage of cells at each staining intensity level was recorded. The H-scores were calculated by following formula:

$$\begin{aligned} {\text{H-score}} & = 0 \, \times \, \left( {\% {\text{ cells }}\left[ 0 \right]} \right) \, + { 1 } \times \, \left( {\% {\text{ cells }}\left[ 1\right]} \right) \, \hfill \\ & \quad + { 2 } \times \, \left( {\% {\text{ cells }}\left[ 2\right]} \right) \, + { 3 } \times \, \left( {\% {\text{ cells }}\left[ 3\right]} \right). \hfill \\ \end{aligned}$$

### Bioinformatics

Ingenuity Pathway Analysis software (IPA, Qiagen, Inc. Redwood City, CA, USA) was used for bioinformatic analysis of networks involving the biological relationship between YAP1 and pancreatic cancer. A network involving all direct interactors of these proteins was built and analyzed for pathway enrichment and functional annotations.

### Cell culture

The patient derived pancreatic cancer cell line Panc-1 (ATCC-LGC Standards, Manassas, VA, USA) was used for the in vitro experiments. The cells were maintained in DMEM supplemented with 10% fetal bovine serum, 100 U/ml penicillin and 100 μg/ml streptomycin and kept in a humified atmosphere, in 5% CO_2_ at 37 °C. Prior experiment, the cells were observed using phase contrast microscope to ensure the condition of the cells including morphological characteristics and vitality.

### Immunofluorescence based Cellomics

To assert the YAP1 expression profile, the cells were seeded in 6 well plates with the density of fifty thousand cells per well. After 48 h, the cells were fixed with 4% paraformaldehyde (Histolab, Västra Frölunda, Sweden) and stained with primary rabbit anti-human YAP1 (dilution 1: 250, Cell Signaling) followed by Alexa Fluor 488 conjugated donkey-anti-rabbit secondary antibody (dilution 1:200, Invitrogen, USA). The nucleus was marked using DAPI (NucBlue^®^, Molecular probes, Life technologies, USA). Cellomics ArrayScan platform VTI HCS (ThermoScientific, Rockford, IL, USA) reader connected to Bioapplication software was thereafter used for image processing.

In each well, a cell population consisting of two thousand cells was analysed using multiparameter fluorescent microscopic imaging system designed for high content screening. The processed data obtained from automatically acquired images were quantified as fluorescence intensity for the selected channel (Alexa 488). The accessed images were visualized using automated fluorescence microscopy.

### YAP1 target gene expression

To evaluate the expression of selected YAP1 target genes, the cells were seeded in 6-well plates with a concentration of thirty thousand cells per well. After one cell cycle, the cells were incubated with a maximal tolerable dose of three substances interrupting YAP–TEAD interaction; Super-TDU (500 nM), Verteporfin (100 nM) and CA3 (100 nM) or complete medium. After 48 h, the cell lysates and conditioned medium from respective well and plate were collected. All experiments were executed in triplicates. Expression levels of YAP1 targets genes, including amphiregulin (AREG), connective tissue growth factor (CTGF), cysteine-rich angiogenic inducer 61 (CYR61), fibroblast growth factor 1 (FGF1) and mesothelin (MSLN), were selected from the Ingenuity Pathway Analysis and measured in each sample using enzyme-linked immunosorbent assay (ELISA). 100 µg protein from respective sample was analyzed in each assay according to the manufacturer’s instructions. AREG, CTGF, CYR61, FGF1 were purchased from Nordic Biosite AB, Täby, SE and MSLN from Biolegend, San Diego, CA, USA.

### Statistical analysis

The correlation between YAP1 expression levels and clinicopathological parameters was estimated using the Mann–Whitney U test for continuous variables and Fisher’s exact test or χ^2^ for categorical variables. The Kaplan–Meier method was used to model the cumulative probability of overall survival (OS) and disease-free survival (DFS) and statistical differences were assessed using the log-rank test. Univariable and multivariable survival analysis were also performed using Cox proportional hazards regression modeling.

One-way ANOVA parametric test was applied to compare the concentrations of secreted YAP target genes measured in condition medium obtained from Panc-1 cells subjected to three substances interrupting YAP1 transcriptional activity or untreated cells.

Statistical evaluation was conducted with SPSS version 23.0 (SPSS Inc., Chicago, IL, USA) and GraphPad Prism v.8.0.1 (La Jolla, CA, USA). A p-value < 0.05 was considered statistically significant.

## Results

### YAP1 is the top upregulated protein in pancreatic cancer

**Fig. 1:**
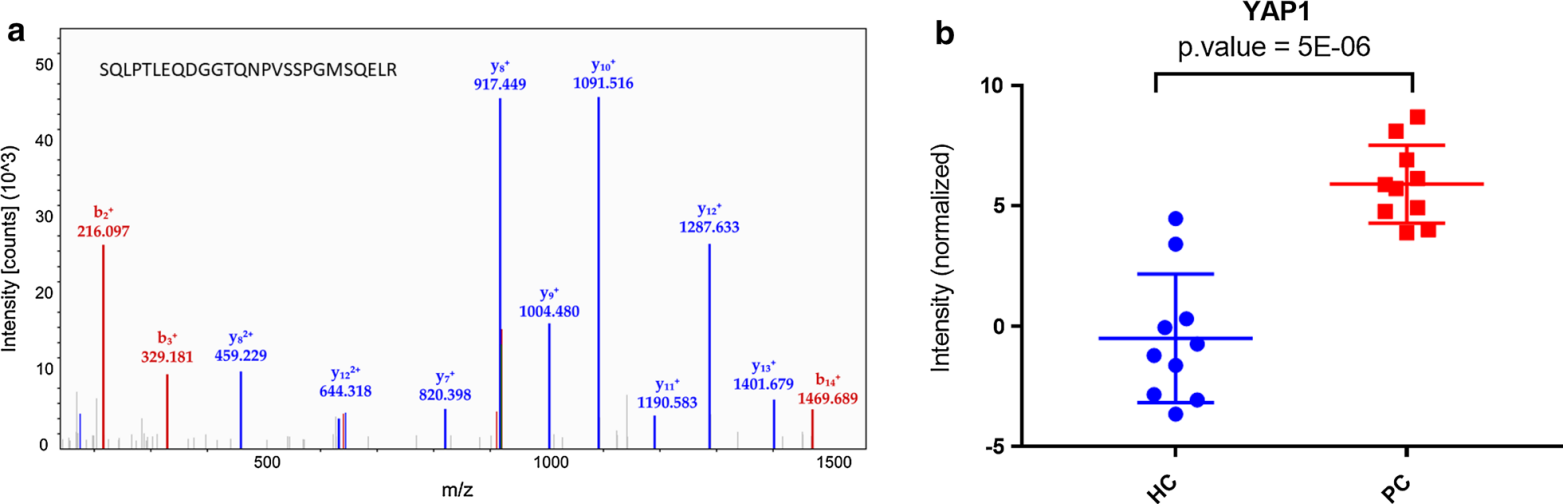
Selection of the YAP1 protein for validation. **a** Label-free quantitative MS spectra of YAP1 (based on peptide SQLPTLEQDGGTQNPVSSPGMSQELR). **b** Box-plot showing relative expression levels of YAP1 in pancreatic cancer (PC) and healthy controls (HC)

Fresh frozen biopsies from pancreatic tumors (n = 10) and healthy pancreatic tissue (n = 10), were analyzed using label-free quantitative proteomics to discover differentially expressed proteins. In total, 4138 proteins were identified, and 2950 proteins were quantified based on one or more unique peptides. 165 candidates were subsequently determined as potential biomarkers for pancreatic cancer, as previously reported [10]. Characterized by six unique peptides, YAP1 was annotated as the top upregulated protein in pancreatic tumor specimens (log2 fold change 6.4; p = 5E−06) (Fig. 1a, b).

### mRNA expression levels of YAP1 as a prognostic marker

**Fig. 2:**
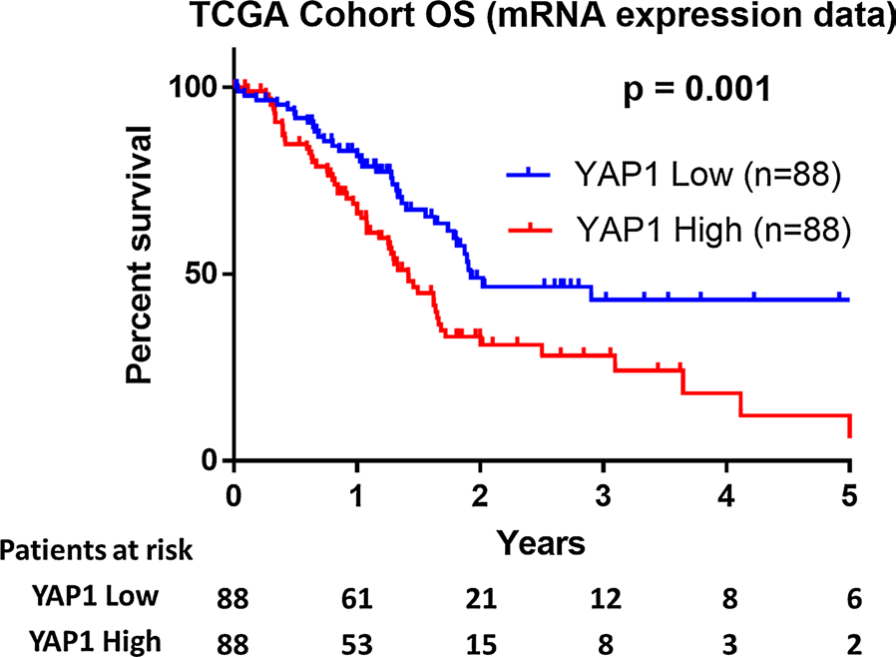
Kaplan–Meier survival curves stratified by YAP1 mRNA expression levels in the TCGA cohort. Patients were categorized based on the median number of fragments per kilobase of exon per million reads (FPKM) into low expression (≤ 19) and high expression groups (> 19)

To assess the prognostic significance of YAP1, we analyzed mRNA expression level data and patient survival based on 176 pancreatic cancer patients included in TCGA (Table 1). The median FPKM value was 19.0, ranging from 0.5 to 46.6. The median FPKM value was used to divide the cohort into a low (FPKM ≤ 19) and a high expression group (FPKM > 19). The Kaplan–Meier plots revealed that high YAP1 mRNA expression was significantly correlated with poorer OS when compared with low mRNA YAP1 expression, as illustrated in Fig. 2 (median survival 17 months vs. 23 months, respectively, p = 0.001).

**Table 1:** Characteristics of the TCGA cohort (n = 176)

Variable	N = 176
Median age (range), years	65 (35–88)
Female gender	50 (45.5%)
AJCC-stage	
I	21 (11.9%)
II	145 (82.4%)
III	3 (1.7%)
IV	4 (2.3%)
Unknown	3 (1.7%)
Median FPKM (range)	19.0 (0.5-46.6)

*FPKM* fragments per kilobase of exon per million reads

### YAP1 protein expression levels and prognosis

**Fig. 3:**
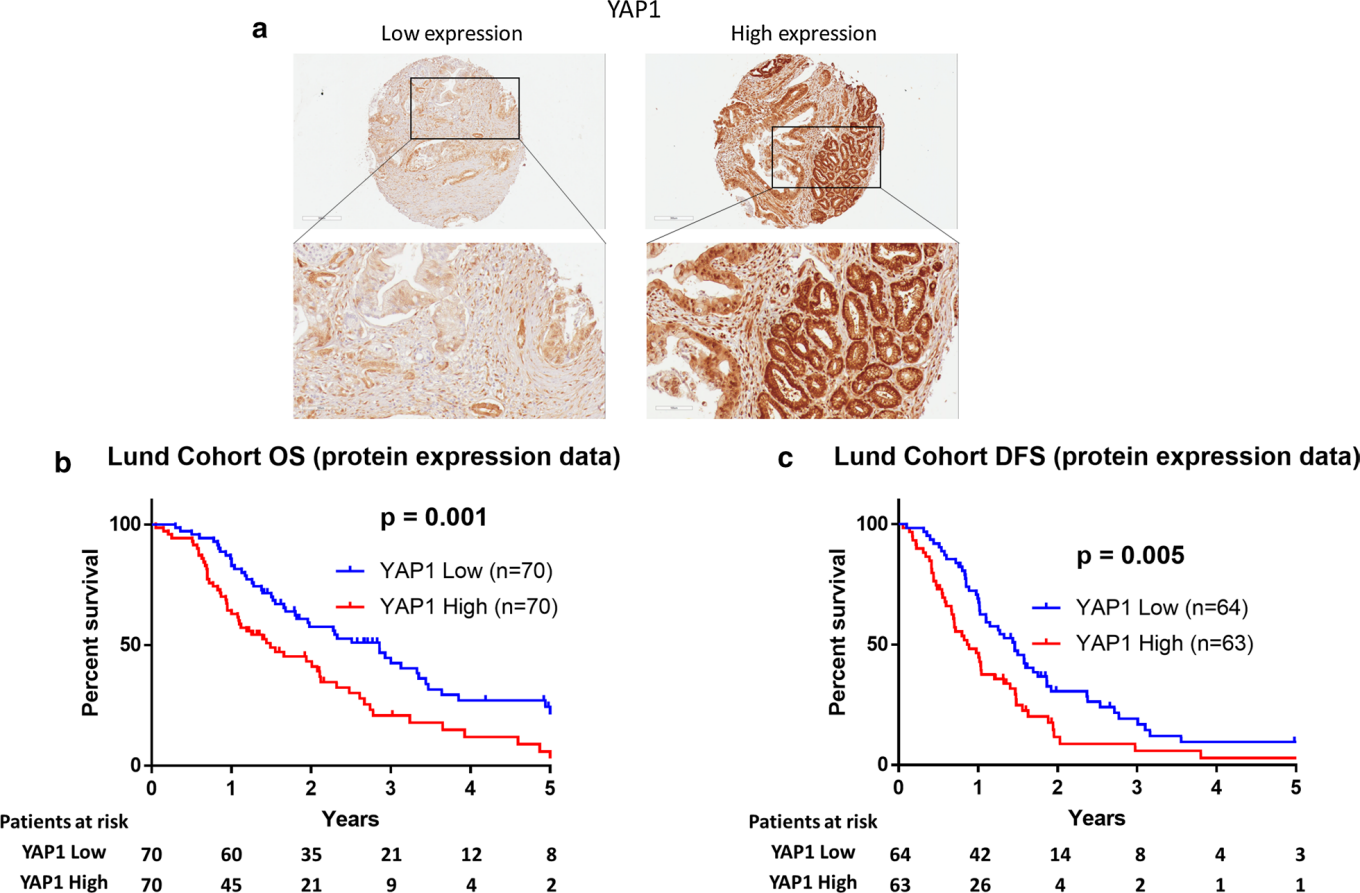
Immunohistochemical analysis of YAP1 protein expression in the tissue microarray cohort. **a** Representative images of YAP1 immunostaining in low and high expression groups using the median H-score (170) as cut-off. **b** Kaplan–Meier survival curves for overall survival stratified by YAP1 protein expression. **c** Kaplan–Meier survival curves for disease-free survival stratified by YAP1 protein expression

The protein expression levels of YAP1 were analyzed using immunohistochemistry staining on TMA sections constructed from 140 pancreatic tumors. The antibody staining specific for YAP1 was detected in the nucleus or in the nucleus and cytoplasm of tumor cells. The median H-score was 170 (range, 59–289). Based on the median H-score (170), a low (H-score ≤ 170) and a high expression group (H-score > 170) were created (Fig. 3a). No significant differences in clinicopathological features were identified between high and low YAP1 expression groups (Table 2).

**Table 2:** Characteristics of the TMA cohort (n = 140)

Variable	N	All patients (n = 140)	Low YAP1 protein expression (n = 70)	High YAP1 protein expression (n = 70)	*p*
Age > 65 years	140	93 (66.4)	48 (68.6)	45 (64.3)	0.721
Female gender	140	66 (47.1)	35 (50)	31 (44.3)	0.612
BMI > 25 kg/m^2^	132	57 (43.2)	32 (47.1)	25 (39.1)	0.383
Smoking history	139	67 (48.2)	28 (40.6)	39 (55.7)	0.09
Diabetes mellitus	139	33 (23.7)	19 (27.1)	14 (20.3)	0.426
Symptoms at diagnosis	136	131 (96.3)	68 (100)	63 (92.6)	0.058
Tumor location (head)	140	117 (83.6)	62 (88.6)	55 (78.6)	0.17
Tumor size > 2 cm	139	117 (84.2)	60 (87)	57 (81.4)	0.487
T-stage ≥ T2	139	121 (87.1)	60 (87)	61 (87.1)	1
N-stage ≥ N1	138	104 (75.4)	53 (76.8)	51 (73.9)	0.844
AJCC-stage ≥ II	138	112 (81.2)	56 (81.2)	56 (81.2)	1
Histological grade ≥ 3	138	83 (60.1)	38 (55.9)	45 (64.3)	0.385
Positive resection margin	139	55 (39.6)	28 (40.6)	27 (38.6)	0.863
Adjuvant chemotherapy	135	113 (83.7)	60 (87)	53 (80.3)	0.355
Recurrence of disease	127	103 (81.1)	51 (79.7)	52 (82.5)	0.821

N, number of non-missing values. Qualitative data are expressed as n (%)

*AJCC* American Joint Committee on Cancer, *BMI* body mass index, *N-stage* nodal stage, *T-stage* tumor stage

Kaplan–Meier analysis revealed that high YAP1 protein expression was significantly correlated with shorter OS when compared with low YAP1 protein expression (median survival, 17.9 vs. 34.3 months, respectively, p = 0.001, log-rank test; Fig. 3b). Furthermore, patients exhibiting high YAP1 protein expression had significantly reduced DFS when compared to the low YAP1 protein expression group (median DFS, 10.7 vs. 17.5 months, respectively, p = 0.005, log-rank test; Fig. 3c).

The univariable Cox regression analysis of OS identified smoking history (p = 0.04), symptoms at diagnosis (p = 0.05), histopathological grade (p = 0.03), and high expression of YAP1 (p = 0.001) as factors associated with shorter OS. In multivariable Cox regression analysis, high YAP1 protein expression was identified as an independent risk factor for poor OS (hazard ratio (HR) 1.870, 95% confidence interval (CI) 1.224–2.855, p = 0.004). Moreover, univariable Cox regression analysis of DFS determined histopathological grade (p = 0.028), resection margin ≥ R1 (p = 0.028), and high expression of YAP1 (p = 0.006) as factors associated with decreased DFS. Multivariable Cox regression analysis confirmed the results, indicating that high YAP1 protein expression is an independent risk factor for reduced DFS (HR 1.950, 95% CI 1.299–2.927, p = 0.001) (Table 3).

**Table 3:** Univariable and multivariable Cox regression analysis in the TMA cohort (n = 140)

Variable	OS				DFS			
	Univariable HR (95% CI)	*p*	Multivariate HR (95% CI)	*p*	Univariable HR (95% CI)	*p*	Multivariable HR (95% CI)	*p*
Age (> 65)	0.994 (0.658–1.501)	0.977			0.760 (0.506–1.144)	0.189		
Female gender	0.825 (0.557–1.221)	0.336			0.675 (0.453–1.005)	0.053		
BMI (> 25 kg/m^2^)	1.250 (0.832–1.876)	0.283			1.372 (0.913–2.061)	0.128		
Smoking history	1.510 (1.019–2.239)	0.04*	1.319 (0.868–2.003)	0.195	1.268 (0.852–1.887)	0.242		
Diabetes	0.782 (0.479–1.277)	0.326			0.927 (0.567–1.515)	0.762		
Symptoms at diagnosis	0.363 (0.132–1.000)	0.05*	0.548 (0.193–1.559)	0.260	0.620 (0.227–1.693)	0.351		
Tumor location (head)	0.658 (0.390–1.112)	0.118			1.143 (0.625–2.092)	0.664		
Tumor size (> 2 cm)	1.090 (0.653–1.819)	0.741			1.215 (0.710–2.079)	0.478		
T-stage (≥ T2)	1.152 (0.672–1.973)	0.607			1.429 (0.795–2.571)	0.233		
N-stage (≥ N1)	1.474 (0.924–2.352)	0.104			1.316 (0.829–2.088)	0.244		
AJCC-stage (≥ II)	1.426 (0.855–2.379)	0.174			1.345 (0.814–2.222)	0.248		
Histological grade (≥ 3)	1.580 (1.045–2.390)	0.03*	1.728 (1.123–2.657)	0.013*	1.592 (1.050–2.413)	0.028*	1.628 (1.072–2.472)	0.022*
Resection margin (≥ R1)	1.388 (0.926–2.080)	0.112			1.585 (1.050–2.394)	0.028*	1.716 (1.127–2.613)	0.012*
Adjuvant chemotherapy	0.712 (0.435–1.166)	0.177			1.632 (0.887–3.002)	0.115		
YAP1 protein expression (High)	1.917 (1.288–2.854)	0.001*	1.870 (1.224–2.855)	0.004*	1.752 (1.178–2.608)	0.006*	1.950 (1.299–2.927)	0.001*

Variables with p ≤ 0.05 were marked with asterisk (*), variables with p ≤ 0.05 in univariable analysis were included in multivariable analysis

*AJCC* American Joint Committee on Cancer, *BMI* body mass index, *CI* confidence interval, *DFS* disease free survival, *HR* hazard ratio, *N-stage* nodal stage, *OS* overall survival, *T-stage* tumor stage

We thus interpret that YAP1 may function as a marker for poor prognosis and disease relapse in pancreatic cancer patients.

### YAP1 is connected to mediators promoting remodeling of the extracellular matrix

**Fig. 4:**
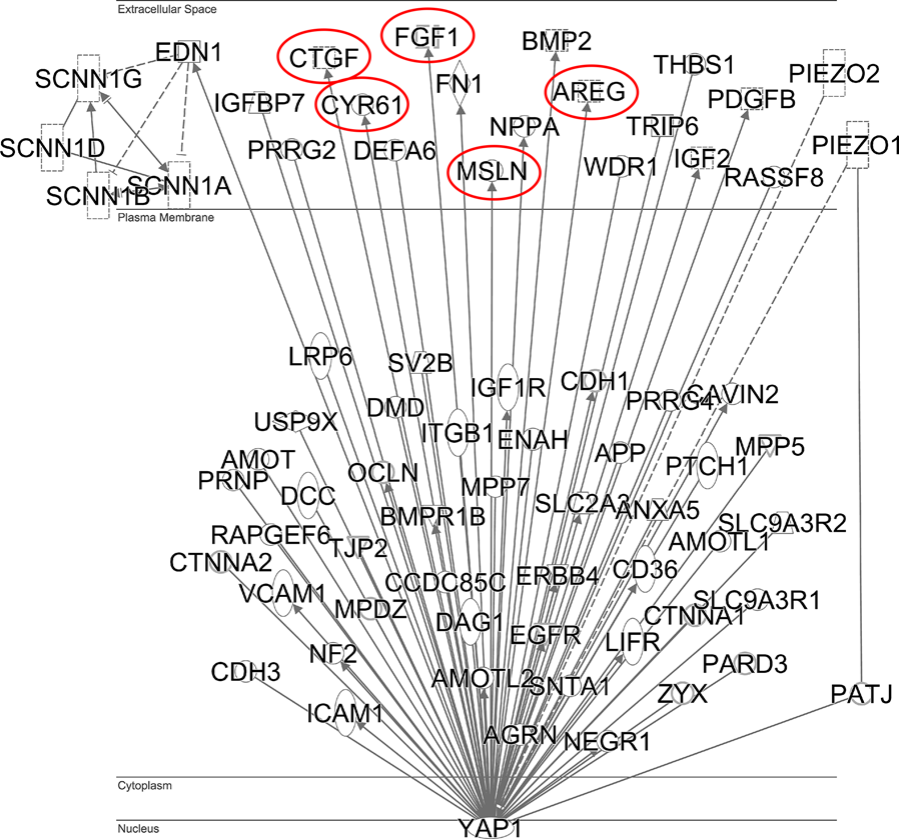
Ingenuity Pathway Analysis showing the plasma membrane and extracellular proteins directly related to YAP1. The relation to proteins involved in mechanotransduction include the cell membrane protein PATJ (crumbs cell polarity complex component), which is directly related to YAP1 and is also interacting with PIEZO1, the Piezo type mechanosensitive ion channel component 1. YAP1 is also an indirect regulator of both PIEZO1 and PIEZO2. Further, the cytokine endothelin 1 (EDN1) is directly related to YAP1 and is also a regulator of the degenerin/epithelial sodium channels (DEG/ENaC, here marked as SCNN1A, SCNN1B, SCNN1G, SCNN1D). Tight junction signaling proteins related to YAP1 include CTNNA1, MPDZMPP5, OCLN, PATJ, TJP2. Epithelial adherens junction signaling proteins related to YAP1 include CDH1, CTNNA1, CTNNA2, EGFR, FGF1, PARD3, ZYX. Examples of secreted proteins involved in creating a pro-fibrotic microenvironment include AREG, CTGF, CYR61, FGF1, and MSLN and these YAP1 target genes are highlighted and were chosen for further in vitro confirmation

Subsequently, we explored the biological background of the obtained results with the aim to identify the most significant networks and relationships associated with YAP1 expression in pancreatic cancer. Bioinformatic analysis using the IPA software revealed that YAP1 is directly related to proteins involved in mechanotransduction, such as PATJ and PIEZO1, and the cytokine EDN1 (Fig. 4). Tight junction signalling proteins related to YAP1 include CTNNA1, MPDZMPP5, OCLN, PATJ, and TJP2, while epithelial adherens junction signaling proteins related to YAP1 include CDH1, CTNNA1, CTNNA2, EGFR, FGF1, PARD3, and ZYX. Examples of secreted proteins involved in creating a pro-fibrotic microenvironment include AREG, CTGF, CYR61, FGF1, and MSLN and these YAP1 target genes were chosen for further in vitro confirmation.

### YAP1 protein expression in a patient derived cell line

**Fig. 5:**
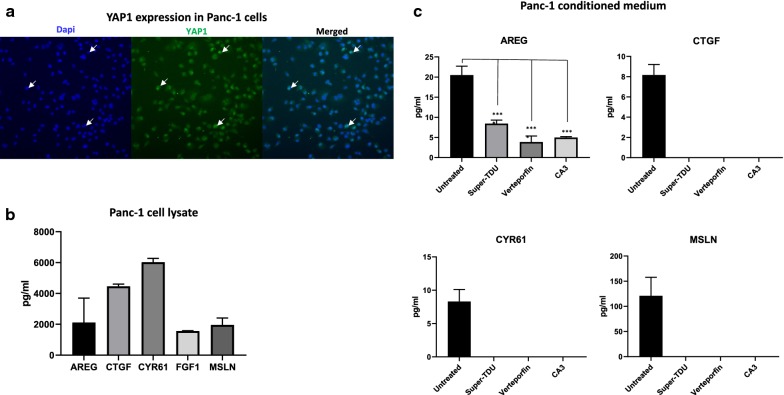
In vitro analysis of YAP1 and selected target genes in Panc-1 cells. **a** YAP1 protein expression in Panc-1 cells. The image represents an immunofluorescence staining of endogenous YAP1 in Panc-1 cells, plated in 6 well plates and cultivated for 48 h under standard conditions. The arrows indicate an exemplification of YAP1 nuclear accumulation. **b** Concentrations of YAP1 target genes in lysates obtained from Panc-1 cells cultivated under standard conditions. C) Concentrations of YAP1 target genes in conditioned medium obtained from Panc-1 cells that were subjected to maximal tolerable doses (MTD) of substances blocking the YAP1/TEAD interaction

We performed immunofluorescence based Cellomics to evaluate the protein expression profile of YAP1 in Panc-1 cells. In accordance with the TMA/IHC patient data, a positive YAP1 staining was detected in both nucleus and cytoplasm of Panc-1 cells. The majority of positively stained cells showed a strong fluoresce intensity located in the nucleus (Fig. 5a).

### YAP1 participates in the transcription of target genes involved in profibrotic tumor microenvironment

Next, we investigated co-transcriptional activity of YAP1 in synthesis of secreted proteins associated with remodeling of the tumor microenvironment in pancreatic cancer. First, Panc-1 cells were cultured under standard conditions to assess the expression levels of proteins ascertained by the IPA analysis. All investigated proteins, AREG, CTGF, CYR61, FGF1, and MSLN were considered as low abundant and detected in low concentrations (pg/ml) in lysates of Panc-1 cells cultured under standard conditions. As presented in Fig. 5b, the expression levels corresponded to at a maximum 0.2‰ of the total cellular protein amount.

Next, the collected conditioned medium from the Panc-1 cells was analyzed for the presence of selected proteins. AREG, CTGF, CYR61, and MSLN were identified and the secretion pattern was further investigated. Panc-1 cells were subjected to substances inhibiting YAP1 transcriptional activity and the concentrations of the determined secreted proteins were measured. Levels of secreted AREG, CTGF, CYR61, and MSLN were significantly lower (p = 0.0001) or undetectable in conditioned medium after the treatment (Fig. 5c). Based on the obtained results, we suggest that YAP1 is involved in the transcription of genes associated with remodeling of the pancreatic tumor microenvironment.

## Discussion

In this transcriptome- and proteome-based study, we identified YAP1 as an indicator of poor OS and DFS in patients with pancreatic cancer.

The American Joint Committee on Cancer (AJCC) tumor-node-metastasis (TNM) classification system is currently the gold standard for pancreatic cancer prognostication [23]. However, the AJCC TNM system is only concerned with the anatomical extent of the disease though patients within the same stage may exhibit different outcomes [24]. Such evaluation may lead to either over- or undertreatment. Improved staging systems, considering molecular factors are necessary in order to enhance individual prognostication and utilization of precision therapies.

The prognostic significance of YAP1 protein expression has only been evaluated in one previous small study by Allende et al. [25]. However, YAP1 protein expression did not reach statistical significance in their Kaplan–Meier analysis, likely due to the small cohort size (64 patients). Only when conducting subgroup analyses, stratifying survival into groups of patients surviving more than or less than 30 months, it was shown that patients with high YAP1 expression had worse survival. Therefore, to clarify the prognostic role of YAP1 protein expression in pancreatic cancer, additional studies based on larger cohorts are needed. The TMA/immunohistochemistry analysis based on 140 patients in our study revealed that overexpression of YAP1 is an independent factor for unfavorable outcome and disease recurrence. These findings are in agreement with the public mRNA dataset from the TCGA, which illustrate that high expression of YAP1 significantly correlates with poor survival in pancreatic cancer patients. The agreement between the transcriptome- and proteome-based survival analyses in the present study strengthens the clinical significance of YAP1 as a prognostic variable. However, it is important to note that knowledge about mRNA abundances can only partially predict protein abundances, with a large fraction of the variance also being explained by other factors such as post-transcriptional and translational regulation, as well as protein degradation [26].

To understand the biological role of YAP1 in pancreatic cancer, we performed bioinformatic analyses of protein networks. The results revealed that YAP1 is directly connected to secreted AREG, CTGF, CYR61, FGF1 and MSLN that are involved in fibrosis and other key signaling pathways involved in the tumor-stroma interactions [27–31].

Pancreatic cancer progression is generally associated with a dense fibrotic stroma characterized by an extensive deposition of extracellular matrix components surrounding the cancer cells [32, 33]. The desmoplastic extracellular matrix, mainly produced by activated cancer associated fibroblasts, accounts for up to 80% of entire tumor mass [33]. The fibrotic environment is known to undergo an extensive remodeling connected to the stiffening of tumor tissue. Such stromal reshaping presumably modifies the crosstalk between residual cells within the tumor and directs the tumor progression towards an aggressive phenotype [33–35]. The increased stiffness of matricellular tumor microenvironment also activates YAP1 to further modulate the behavior of cancer cells on the transcriptional level [36, 37].

YAP1 itself, however, lacks DNA-binding activity and requires an interaction with DNA-binding transcription factors such as TEAD to activate target genes [38]. AREG, CTGF and CYR61 account for the most acknowledged target genes for YAP1/TEAD [39–41]. The YAP1/TEAD interactions are also reported to regulate the expression of FGF1 and MSLN [42–44].

We hypothesized that the secreted YAP1/TEAD target gene products contribute to the enhanced fibrotic reaction and intra-tumoral stiffening which consecutively promote YAP1 transcriptional activity. Such paracrine loop would further affect the tumor microenvironment and maintain the aggressive course of the disease.

Using the patient derived pancreatic cancer cell line Panc-1, we evaluated the effect of substances designed to inhibit the YAP1/TEAD mediated gene transcription. We showed that the disruption of YAP1/TEAD complex significantly reduced the presence of the selected YAP1/TEAD target gene products in the conditioned medium. Suppression of YAP1 oncogenic activity with a subsequent modification of the tumor microenvironment may thus be an advantageous approach to control tumor growth and improve prognosis. Although the clinical utilization for such treatment remains to be determined, YAP1 as a biomarker may aid in the individual prognostication of patients diagnosed with pancreatic cancer and the selection of precision therapy.

## Conclusions

We demonstrate that YAP1 is an independent prognostic marker associated with recurrence and unfavorable survival in pancreatic cancer. We also show that inhibition of YAP1/TEAD interaction interferes with the expression of AREG, CTGF, CYR61, and MSLN suggesting that YAP1 transcriptional activity may affect the development and persistence of a fibrotic tumor microenvironment. YAP1 is thus considered as a clinically and biologically relevant biomarker derived from pancreatic cancer tissue.

## Data Availability

The datasets generated and/or analyzed during the current study are available from the corresponding author on reasonable request.

## Abbreviations

AJCC: American Joint Committee on Cancer
AREG: Amphiregulin
BMI: Body mass index
CI: Confidence interval
CTGF: Connective tissue growth factor
CYR61: Cysteine-rich angiogenic inducer 61
DFS: Disease-free survival
ELISA: Enzyme-linked immunosorbent assay
FFPE: Formalin-fixed paraffin-embedded
FGF1: Fibroblast growth factor 1
FPKM: Fragments per kilobase of exon per million reads
HR: Hazard ratio
IHC: Immunohistochemistry
IPA: Ingenuity Pathway Analysis
MS: Mass spectrometry
MSLN: Mesothelin
OS: Overall survival
TCGA: The Cancer Genome Atlas
TMA: Tissue microarray
TNM: Tumor-node-metastasis
YAP1: Yes-associated protein 1
